# GLP-1 Receptor Agonists and Fertility: What Is Known So Far?

**DOI:** 10.5935/1518-0557.20260028

**Published:** 2026

**Authors:** Alice Scalzilli Becker, Júlia Prauchner de Castilhos, Sophia Abur Said Shugair, Talita Colombo, Marta -Ribeiro Hentschke

**Affiliations:** 1 School of Medicine, Pontifical Catholic University of Rio Grande do Sul, Porto Alegre, Rio Grande do Sul, Brazil; 2 Service of Gynecology and Obstetrics, Santa Casa de Porto Alegre, Porto Alegre, Rio Grande do Sul, Brazil; 3 Fertilitat, Center for Reproductive Medicine, Porto Alegre, Rio Grande do Sul, Brazil

**Keywords:** GLP-1 receptor agonists, fertility, obesity, PCOS, reproductive health

## Abstract

GLP-1 receptor agonists (GLP-1RAs) have gained attention as a potential adjunct in preconception care for women with polycystic ovary syndrome (PCOS) and infertility. This narrative literature review (June 2025) searched PubMed, SciELO, and LILACS using the descriptors “weight loss medication,” “GLP-1 receptor agonists,” “liraglutide,” “semaglutide,” “fertility,” “PCOS,” and “reproductive outcomes,” including studies published between 2014 and 2025, yielding 47 relevant articles. Overall, GLP-1RAs show consistent metabolic benefits and emerging evidence of improved reproductive outcomes in PCOS, with clinical trials reporting increased menstrual regularity, ovulation, and pregnancy rates-particularly when combined with metformin. Liraglutide (1.2-3.0 mg/day) and exenatide (10 µg twice daily) have been associated with improved follicular development and endometrial receptivity. Mechanistically, GLP-1 receptors are expressed in reproductive tissues, and these agents may exert anti-inflammatory, antifibrotic, and androgen-lowering effects; liraglutide has also been reported to restore granulosa-oocyte communication via suppression of CXCL10. In one randomized trial, pregnancy rates were higher with liraglutide plus metformin (69.2%) than with metformin alone (35.4%; *p*<0.05). Ovulation rates up to 86% have been described with exenatide plus metformin, exceeding those observed with monotherapy. Despite these signals of benefit, GLP-1RAs remain contraindicated during pregnancy due to limited human data and fetal risks observed in animal studies; semaglutide and tirzepatide require an 8-10 week washout period prior to conception, and tirzepatide may reduce oral contraceptive effectiveness because of delayed gastric emptying. In summary, GLP-1RAs are a promising strategy for preconception management in women with PCOS and obesity-related infertility, especially in combination with metformin, but they should be avoided during pregnancy and lactation, and individualized counseling is essential to align therapy with reproductive goals.

## INTRODUCTION

Obesity represents a growing challenge in reproductive medicine and is well-established as a major risk factor for subfertility and adverse reproductive outcomes. Excess adiposity is associated with significant endocrine disturbances, including increased aromatization of androgens to estrogens in adipose tissue, reduced levels of sex hormone-binding globulin (SHBG), and compensatory hyperinsulinemia due to insulin resistance. These mechanisms contribute to a state of hyperandrogenism, impaired folliculogenesis, and ovulatory dysfunction, ultimately exacerbating infertility risk (Pasquali & Gambineri, 2018; Escobar-Morreale, 2018). Among the endocrine disorders linked to infertility, polycystic ovary syndrome (PCOS) is the most prevalent condition affecting women of reproductive age (Burgart, 2020).

While lifestyle modification - comprising nutritional counseling and regular physical activity - remains the first-line recommendation for managing obesity and PCOS, long-term adherence is often suboptimal, and clinical results are frequently disappointing. In a study by Sacha *et al*. (2018), involving 148 women aged ≤45 years with infertility or recent pregnancy loss, 69% were actively attempting weight reduction. However, a considerable proportion of overweight (92%) and obese (84%) participants were unwilling to postpone fertility treatment for more than three months. These findings underscore that, although patients recognize the detrimental impact of excess weight on fertility, many prioritize rapid conception strategies, particularly in the context of advanced maternal age (Sacha *et al*., 2018).

Incretins are gastrointestinal hormones that play a crucial role in glucose homeostasis by enhancing insulin secretion in a glucose-dependent manner. The most studied incretins include glucagon-like peptide-1 (GLP-1) and glucose-dependent insulinotropic polypeptide (GIP). GLP-1 receptor agonists (GLP-1RAs) are synthetic analogs of GLP-1 designed to mimic its effects, promoting insulin secretion, inhibiting glucagon release, delaying gastric emptying, and inducing satiety, thereby contributing to weight loss. Several GLP-1RAs are clinically available, including dulaglutide, semaglutide, liraglutide (LIRA), and exenatide (EXE), which differ in molecular structure, half-life, and route of administration. These agents can be classified as short-acting (e.g., EXE, lixisenatide) or long-acting (e.g., LIRA, dulaglutide, semaglutide), which influences their glycemic and weight-lowering effects (Kalra *et al*., 2021).

Beyond glycemic control, GLP-1RAs have shown potential benefits in reproductive health, particularly among women with obesity or PCOS, by improving insulin sensitivity and supporting weight reduction. Against this backdrop, GLP-1RAs have emerged as promising therapeutic agents due to their combined metabolic and potential reproductive benefits. Their use offers novel avenues for women with PCOS or infertility - especially those with diminished ovarian reserve or advanced reproductive age - who cannot afford to delay fertility treatment while awaiting substantial metabolic improvement (Kalra *et al*., 2021).

Currently, three GLP-1RAs are approved by the U.S. Food and Drug Administration (FDA) specifically for weight management: Saxenda® (LIRA), Wegovy® (semaglutide), and Zepbound® (tirzepatide). In addition, Ozempic® (semaglutide), Rybelsus® (oral semaglutide), and Mounjaro® (tirzepatide) are indicated for the treatment of type 2 diabetes mellitus (T2DM), though they are frequently prescribed off-label for weight reduction.

Gynecologists and obstetricians are uniquely positioned to manage obesity in women seeking fertility, as they are often the sole healthcare providers consulted during the reproductive years. Their proactive engagement may enhance the acceptance and continuity of pharmacological weight-loss strategies, such as GLP-1RAs, ultimately improving both metabolic control and fertility outcomes (Gill & MacKey, 2021).

The aim of this review is to provide a comprehensive and critical synthesis of the current evidence regarding the use of GLP-1RAs in women with PCOS and infertility, with a focus on fertility-related outcomes. To achieve this, we compiled and organized all relevant studies addressing GLP-1RA use in this population, including information on dosage, duration of treatment, and expected reproductive outcomes. By offering practical and up-to-date guidance, this review seeks to assist clinicians in the appropriate selection and counseling of patients who may benefit from these agents, while outlining realistic expectations regarding reproductive success and supporting evidence-based decision-making in preconception care.

## MATERIALS AND METHODS

A narrative review was conducted independently by two reviewers on three electronic databases - Public Medline (PubMed), Scientific Electronic Library Online (SciELO), and Latin American and Caribbean Health Sciences Literature (LILACS). The search had no initial date restriction and was performed up to June 2025 using the keywords: “weight loss medication,” “medical therapies for weight loss,” “GLP-1 Receptor Agonists,” liraglutide, semaglutide, fertility, infertility, reproductive health outcomes, combined with the Boolean operators “OR/AND” as appropriate, resulting in a total of 47 articles.

Duplicates were identified and excluded. Two researchers (A.S.B. and J.P.C.) independently evaluated titles and abstracts against the inclusion and exclusion criteria. Publications for which the information needed could not be extracted from the title or abstract were selected for full article reading. All studies selected in the first evaluation were referred for analysis of the full text. Disagreements were resolved by arriving at a consensus in discussions or by a third researcher (S.A.S.S.).

The inclusion criteria for this narrative review comprised studies involving women with PCOS and infertility, with or without obesity, who received treatment with GLP-1RAs such as LIRA, semaglutide, EXE, or tirzepatide. Eligible studies reported reproductive outcomes, including ovulation rates, menstrual regularity, pregnancy rates, follicular development, endometrial receptivity, and relevant hormonal or molecular markers. There were no exclusions based on language, study type, or animal studies, given the limited number of publications on this emerging and recent topic.

**Figure 1:**
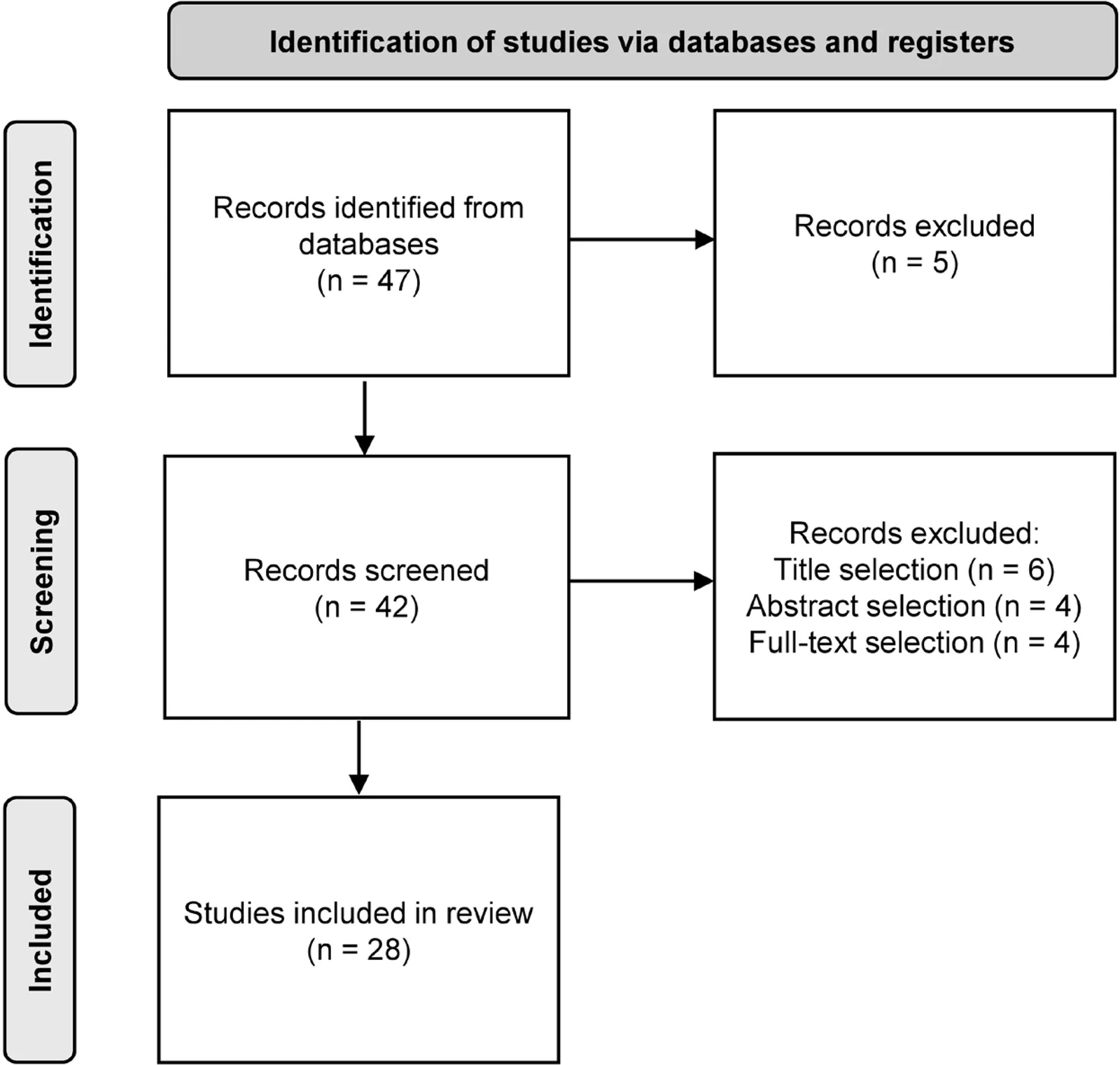
Flowchart of the identification and selection of studies through database searches.

The articles were screened by title and abstract according to the inclusion criteria. After title screening, 5 articles were excluded; following abstract review, an additional 6 articles were excluded. The full texts of the remaining selected documents were then independently evaluated by two reviewers. After full-text review, 4 articles were excluded for lacking specific data on the use of GLP-1RAs. According to the exclusion criteria, 4 studies were further excluded due to unavailability of full-text access (Figure 1 and Table 1).

**Table 1:** Studies included for analysis

Author (Year)	Study Design (Analysis Period)	Language (Country)	Studied Population
Abdalla *et al*. (2021)	Literature review	English	Women with PCOS
Ammar *et al*. (2023)	Literature review	English	Obese men
Bednarz *et al*. (2022)	Literature review	English	Obese women with PCOS
Cena *et al*. (2020)	Literature review	English	Obese women with PCOS
Cesta *et al*. (2024)	Observational cohort (2009-2021)	English (Finland, Iceland, Norway, Sweden, Israel, USA)	Pregnant women with type 2 diabetes
Du Plessis *et al*. (2024)	Literature review	English	Men using GLP-1RAs
Duah & Seifer (2025)	Literature review	English	Overweight or obese reproductive-age women
Fontoura *et al*. (2014)	Case report (2011)	English (Brazil)	35-year-old man
Elkind-Hirsch *et al*. (2021)	Randomized clinical trial (32 weeks)	English	Obese women with PCOS
Elkind-Hirsch *et al*. (2008)	Randomized clinical trial (32 weeks)	English	Overweight/obese women with PCOS
Gill & Mackey (2021)	Literature review	English	Obese women
Gregorič *et al*. (2025)	Open-label randomized clinical trial (2020-2023)	Slovenian (English)	Men with type 2 diabetes and functional hypogonadism
Jensterle *et al*. (2019)	Literature review	English	GLP-1RAs and fertility
La Vignera *et al*. (2023)	Clinical trial	Italian (English)	Obese men with hypogonadism
Liu *et al*. (2017)	Randomized clinical trial (24 weeks)	English (China)	Overweight or obese women with PCOS
Maslin *et al*. (2024)	Literature review	English	Overweight or obese women
Merhi (2025)	Literature review	English	Obese women
Minis *et al*. (2023)	Literature review	English	Overweight or obese women with PCOS
Muller *et al*. (2023)	Literature review	English	Overweight or obese women
Nuako *et al*. (2023)	Literature review	English	Reproductive-age obese women
Nylander *et al*. (2017)	Randomized clinical trial (26 weeks)	English	Women with PCOS
Papaetis & Kyriacou (2022)	Literature review	English	Women with PCOS
Salamun *et al*. (2018)	Open prospective study (12 months)	English (Slovenia)	Obese women with PCOS
Service *et al*. (2023)	Systematic literature review (2013-2023)	English	Obese men
Sola-Leyva *et al*. (2025)	Literature review	English	Reproductive-age obese women with PCOS and infertility
Sundararaman *et al*. (2025)	Literature review	English	Adults with obesity, type 2 diabetes, or cardiovascular, renal, and neurological diseases
Zhao *et al*. (2024)	Translational preclinical experimental study	English (China)	Women with PCOS + animal and cellular models

### PRECONCEPTION USE (TABLE 2)

Recent evidence has identified the presence of GLP-1 receptors in the reproductive tract of most mammals, including the hypothalamus, ovaries, testes, and endometrium. Studies indicate that GLP-1RAs exert anti-inflammatory and antifibrotic effects on the ovaries and endometrium, in addition to benefiting conditions such as obesity, T2DM, and PCOS. GLP-1RAs also appear to reverse polycystic ovarian morphology, reduce serum androgen levels, and decrease their bioavailability in women with PCOS (Duah & Seifer, 2025; Jensterle *et al*., 2019).

**Table 2:** Evidence-Based Recommendations for Preconception Use

Reference	Population	Intervention	Comparison	Duration of Use	Expected Outcome
Nylander *et al*., 2017	72 women with PCOS	Liraglutide 1.8 mg/day	Placebo	26 weeks	Improved bleeding pattern; increased SHBG; decreased free testosterone and ovarian volume
Elkind-Hirsch *et al*., 2021	55 women with obesity and PCOS	Liraglutide 3 mg/day	Placebo	32 weeks	Reduction in free androgen index, increased SHBG levels, and higher menstrual frequency
Salamun *et al*., 2018	28 women with obesity and PCOS	Liraglutide 1.2 mg/day + Metformin 1000 mg BID	Metformin 1000 mg BID	12 weeks	Higher IVF pregnancy rate (85.7% vs. 28.6%); higher cumulative pregnancy rate at 12 months (69% *vs*. 36%); occurrence of spontaneous pregnancies
Elkind-Hirsch *et al*., 2008	60 women with overweight / obesity and PCOS	1) Exenatide 10 µg BID; 2) Exenatide 10 µg BID + Metformin 1000 mg BID	Metformin 1000 mg BID	24 weeks	Increased menstrual regularity and ovulation rate (86% with EXE+MET vs. 50% EXE and 29% MET); decreased FAI; increased SHBG
Liu *et al*., 2017	176 women with overweight / obesity and PCOS	Exenatide 10 µg BID	Metformin 1000 mg BID	12 weeks	Higher spontaneous pregnancy rate (EXE 43.6% *vs*. MET 18.7%)

In a meta-analysis and systematic review including 11 randomized controlled trials (RCTs) with 840 patients, Zhou *et al*. (2023) found that GLP-1RAs increased natural pregnancy rates and improved menstrual regularity. There were no statistically significant differences in overall pregnancy rate or in vitro fertilization (IVF) pregnancy rate between groups; however, total pregnancy rate increased shortly after GLP-1RA use based on subgroup analysis. Randomization to GLP-1RA treatment led to significant improvements in homeostasis model assessments for insulin resistance, body mass index (BMI), waist circumference, and SHBG, as well as a slight decrease in total testosterone compared to controls (Zhou *et al*., 2023).

In a study by Elkind-Hirsch *et al*. (2021), 82 women diagnosed with PCOS were randomized to receive LIRA 3 mg/day (n=55) or placebo (n=27) for 32 weeks. Results demonstrated that LIRA administration significantly reduced free androgen index (FAI) and increased SHBG levels compared to placebo (*p*=0.049 and *p*=0.006, respectively). Additionally, menstrual frequency was significantly increased in the LIRA-treated group relative to placebo.

RCTs have addressed pregnancy rates following preconception treatment with GLP-1RAs, both reporting higher pregnancy rates after discontinuation of GLP-1RAs in women with PCOS. Salamun *et al*. conducted an RCT with 27 obese women (mean BMI 36.7±3.5 kg/m^2^) with PCOS; 13 received combined therapy with metformin (MET) 1000 mg twice daily and LIRA 1.2 mg daily, while 14 received MET monotherapy for 12 weeks. After 12 months, pregnancy rate was significantly higher in the combination therapy group (69.2%) compared to MEt alone (35.4%). Moreover, embryo transfer pregnancy rates were significantly increased in the combination group (85.7%) *versus* MET (28.6%; *p*=0.03), suggesting that addition of LIRA may improve endometrial quality and receptivity in PCOS women undergoing IVF. None of the participants had prior weight loss despite lifestyle changes, and all were resistant to first-line reproductive therapies with aromatase inhibitors or clomiphene citrate (Salamun *et al*., 2018). Another RCT involving 72 PCOS women demonstrated that daily subcutaneous LIRA 1.8 mg improved ovarian function, increased menstrual regularity, and reduced androgen levels compared to placebo (Nylander *et al*., 2017).

In line with comparisons between GLP-1RAs and MET, a meta-analysis evaluated 8 RCTs comparing the efficacy of both treatments in women with PCOS. The study concluded that GLP-1RAs were significantly superior to MET in multiple metabolic and reproductive parameters, including improvements in insulin sensitivity, BMI, abdominal circumference, menstrual cyclicity, serum total testosterone, FAI, SHBG, androstenedione, luteinizing hormone (LH), dehydroepiandrosterone sulfate (DHEA-S), Ferriman-Gallwey scores, fasting glucose and insulin, triglycerides, total cholesterol, and blood pressure (Merhi, 2025).

An experimental study by Zhao *et al*. (2024) investigated the effects of LIRA on follicular development and ovulation in women with PCOS. Using a translational approach, the authors combined human patient data and murine models to elucidate underlying cellular mechanisms. They first demonstrated significantly elevated of C-X-C motif chemokine ligand 10 (CXCL10) in follicular fluid and granulosa cells from PCOS women compared to controls. CXCL10 is a pro-inflammatory chemokine, which is involved in the occurrence of various metabolic and inflammatory diseases. They then showed that in vitro CXCL10 supplementation impaired follicular growth, oocyte maturation, and ovulation primarily by destabilizing oocyte-granulosa cell communication via overexpression of GJA1 (encoding connexin 43, a gap junction protein). LIRA treatment restored follicular dynamics by suppressing CXCL10 secretion through inhibition of the Janus kinase - Signal Transducer and Activator of Transcription (JAK-STAT) signaling pathway, thus reducing cytokine and growth factor activity locally. The JAK signaling pathway regulates multiple cellular mechanisms associated with the development of various diseases. These findings indicate that beyond metabolic effects, LIRA exerts local ovarian actions by modulating inflammation and promoting a favorable microenvironment for follicular development and ovulation in PCOS - supporting its potential preconception use in anovulatory infertility (Zhao *et al*., 2024).

Burgart reports that GLP-1RAs such as LIRA are effective in promoting weight loss in PCOS women, contributing to improved metabolic and reproductive parameters associated with the condition. Besides weight reduction, these agents may exert direct and indirect beneficial effects on the hypothalamic-pituitary-ovarian axis, favoring reproductive function (Burgart, 2020). Thus, treatment with LIRA at doses of 1.2 to 3.0 mg promoted menstrual regularization, androgen reduction, and ovulation improvement (Gill & MacKey, 2021).

In a pioneering RCT, 60 overweight or obese PCOS women with oligo-ovulation received 24 weeks of treatment with either MET 1000 mg twice daily, EXE 10 µg twice daily, or combination therapy. Notably, EXE plus MET produced a greater reduction in free androgen index and improvement in menstrual frequency compared to MET monotherapy, findings strongly associated with weight loss (*p*=0.018). Ovulation rates reached 86% in the combination group, surpassing EXE (50%) and MET (29%) groups (*p*<0.01). This was the first study to demonstrate that EXE combined with MET over 24 weeks is more effective than either agent alone for promoting menstrual regularity and ovulation in overweight PCOS women (Elkind-Hirsch *et al*., 2008).

Subsequently, an RCT with 176 overweight or obese PCOS women compared 12 weeks of EXE *versus* MET treatment, followed by 12 weeks of MET for all participants. Those initially treated with EXE showed greater weight loss, improved insulin resistance, and increased menstrual frequency. Additionally, natural pregnancy rates were significantly higher in the EXE pretreated group (43.6% *vs*. 18.7%; *p*<0.05), suggesting that short-term EXE may be more effective than MET in enhancing fertility, possibly due to weight reduction (Liu *et al*., 2017).

Furthermore, an in vivo study demonstrated that EXE reduces histological degeneration and fibrosis of the endometrium in diabetic rats, mainly through anti-inflammatory effects and oxidative stress neutralization. These results suggest that GLP-1RAs may exert anti-inflammatory and antifibrotic properties on the endometrium affected by obesity, diabetes, and PCOS, improving endometrial receptivity in this population (Sola-Leyva *et al*., 2025).

Nonclinical studies with tirzepatide in animal models have reported adverse reproductive effects in females. Rats treated with varying doses during early embryonic development exhibited prolonged diestrus, reduced corpora lutea numbers, and consequent decreases in implantation sites and viable embryos. These effects were considered secondary to tirzepatide’s pharmacologic action, particularly appetite suppression and maternal weight loss. However, in male rats, tirzepatide showed no impact on sperm morphology, mating capability, fertility, or conception. Despite these findings, no clinical data are currently available in humans to clearly establish tirzepatide’s effects on female or male fertility (MOUNJARO Highlights, 2025).

### USE DURING PREGNANCY

The substantial increase in periconceptional use of GLP-1RAs, especially in the United States, reflects a shift in the management of T2DM in women of reproductive age. With the recent approval of these agents for obesity treatment, fetal exposure to this drug class is expected to continue rising. In both Europe and the United States, GLP-1RAs are classified as pregnancy category C by the European Medicines Agency (EMA) and the Food and Drug Administration (FDA) (Maslin *et al*., 2024).

A multinational population-based cohort study involving over 50,000 pregnant women with T2DM and their offspring reported a higher prevalence of major congenital malformations, particularly cardiac defects, compared to the general population (5.3% *vs*. 3.7%). However, no increased risk of malformations was identified following periconceptional exposure to antidiabetic drugs - including GLP-1RAs, sulfonylureas, DPP-4 inhibitors, and SGLT2 inhibitors - compared to insulin use (adjusted RR: 0.95; 95% CI: 0.72-1.26). These findings provide initial evidence supporting fetal safety of these medications when used early in pregnancy, although continuous monitoring and confirmation by future studies are warranted (Cesta *et al*., 2024).

In a systematic review by Muller *et al*., only three case reports involving human pregnancies exposed to GLP-1RAs were found. Despite fetal exposure during organogenesis, neither study demonstrated anatomical abnormalities or maternal-fetal transfer of the medication. Nevertheless, the authors emphasize the limited scientific evidence inherent to case reports and highlight the lack of established safety for GLP-1RAs during pregnancy due to their pharmacological properties, concluding that these agents cannot be recommended during gestation (Greco, 2015; Ivanišević *et al*., 2018; Muller *et al*., 2023; Williams *et al*., 2009).

Another systematic review by Minis *et al*. (2023), which assessed the safety of GLP-1RAs in the preconception period, identified 16 articles, of which 10 were excluded for being reviews or animal studies. Included were 3 case reports of pregnant women who inadvertently continued GLP-1RA therapy during pregnancy (Burlina *et al*., 2022; Greco, 2015; Yang & Wang, 2016), and 2 RCTs involving patients treated with GLP-1RAs preconceptionally (Liu *et al*., 2017; Salamun *et al*., 2018). No adverse gestational or neonatal outcomes were reported.

As of May 31, 2016, Novo Nordisk’s safety database recorded 271 cases of LIRA exposure during pregnancy. Among these, 45.9% resulted in live births without congenital anomalies, 1.8% had congenital anomalies, 34.2% ended in fetal loss, and 18% in pregnancy terminations. These outcomes were comparable to pregnancies exposed to placebo. Although clinical trials reported fetal loss cases in patients treated with Saxenda®, the similar occurrence in the placebo group precludes direct attribution of risk to the drug. Accordingly, the product labeling for LIRA highlights insufficient data in pregnant women to determine the risk of malformations or spontaneous abortion (Saxenda Patient Labeling Review, 2025).

Regarding LIRA, data on its use during human pregnancy remain scarce. Animal studies reported vascular, renal, skeletal, and oropharyngeal malformations in offspring of pregnant rats exposed to LIRA at doses ≥ 0.8 times the human systemic exposure equivalent to 3 mg subcutaneous daily. LIRA has a half-life of approximately 13 hours, and it is recommended to discontinue therapy 10 to 14 days prior to conception (Saxenda Review, 2025).

The effects of semaglutide on human reproductive health are poorly understood, though animal studies indicate risks. Observations include fetal growth restriction, skeletal and visceral malformations, early pregnancy losses, estrous cycle alterations (increased time in estrus), and reduced corpora lutea number, indicating impaired fertility. These findings suggest potential risk to offspring, and semaglutide use is not recommended during pregnancy or lactation. Semaglutide has a significantly longer half-life of approximately one week, implying complete elimination may take 5 to 7 weeks after the last dose, with a total washout period estimated at 8 to 10 weeks. Therefore, manufacturers recommend discontinuation approximately 2 months prior to conception (Highlights SEMAGLUTIDE, 2025).

Similarly, tirzepatide exhibits a half-life of five days, requiring a comparable elimination and washout period, with recommendations to stop therapy two months before attempting conception (Hall *et al*., 2018).

### USE DURING LACTATION

Animal studies have demonstrated that GLP-1RAs, such as EXE, LIRA, semaglutide, and lixisenatide, are excreted into breast milk in variable proportions (ranging from ≤2.5% up to 50% of maternal plasma concentration). Use during late pregnancy and lactation has been associated with reduced fetal and neonatal growth. In humans, there are no studies evaluating the safety of these drugs during lactation. Theoretically, their transfer into human milk is expected to be limited due to their large molecular size, and a portion of the drug may be degraded in the infant’s gastrointestinal tract. Nonetheless, based on animal data, some degree of milk excretion with possible absorption and systemic effects in the infant is anticipated (Muller *et al*., 2023; Wang *et al*., 2017).

Regarding tirzepatide, no studies-animal or human-have assessed its presence in breast milk, its effects on the nursing infant, or on milk production (Highlights MOUNJARO, 2025).

Lastly, the appetite suppression and significant weight loss associated with GLP-1RA use may lead to decreased milk production, potentially adversely affecting infant nutrition and growth. Therefore, it is prudent that women planning to breastfeed should not (re)start GLP-1RA therapy until after weaning (Duah & Seifer, 2025).

### USE AND CONTRACEPTION

Nuako *et al*. (2023) emphasize that women of reproductive age face overlapping stigmas related to both weight and infertility. This combination may prompt financially able women to obtain GLP-1RAs without a medical prescription, raising concerns due to the lack of individualized healthcare supervision. Moreover, no data currently exist regarding the use of these medications without a prescription or the impact of absent professional guidance, including contraceptive counseling.

In this context, Brazil’s National Health Surveillance Agency (Anvisa) issued Normative Instruction No. 360/2025 and RDC Resolution No. 973/2025 in April 2025, requiring GLP-1RA prescriptions to be issued in duplicate, with one copy retained at the pharmacy, effective June 23, 2025, and prescriptions valid for up to 90 days. The Regional Medical Council of Rio Grande do Sul (CREMERS) highlighted this measure in their communications, reinforcing that prescription retention, similar to antibiotics, aims to ensure medical follow-up and prevent inappropriate self-medication - especially in sensitive populations such as women of reproductive age.

Regarding compatibility with contraceptive methods, studies indicate that LIRA and semaglutide do not alter the absorption of oral contraceptives (Maslin *et al*., 2024). Conversely, tirzepatide delays gastric emptying, potentially impairing the absorption of orally administered drugs. Pharmacokinetic studies showed that after a single 5 mg dose of tirzepatide, plasma levels of combined oral contraceptive components were significantly reduced, with a 59% decrease in maximum ethinylestradiol concentration and up to a 66% reduction in norgestimate, along with approximately a 20-23% decreased overall systemic exposure. Additionally, time to maximum plasma concentration was prolonged by 2.5 to 4.5 hours. Given this potential impact on contraceptive efficacy, women using oral contraceptives are advised to employ non-oral contraceptive methods or add barrier contraception for four weeks after initiating tirzepatide treatment and after each dose escalation (Highlights MOUNJARO, 2025).

### USE IN THE MALE POPULATION

GLP-1 hormone receptors are also present in testicular tissues, suggesting possible influence on reproductive function. Thus, the effects of GLP-1RAs may be mediated not only by weight loss but also via direct mechanisms in gonadal tissues, although these pathways require further confirmation (Ammar *et al*., 2023).

Fontoura *et al*. (2014) reported a case of a 35-year-old man who experienced deterioration of spermatogenesis, with reduced sperm concentration and motility after daily use of 0.6 mg LIRA. Seminal parameters normalized five months after discontinuation, suggesting possible adverse effects on fertility. Another study showed that GLP-1 signaling reduced testosterone production and potentially impacted sperm quality (Jeibmann *et al*., 2005).

Conversely, Andersen *et al*. (2022) conducted a randomized, double-blind controlled trial with 56 men including LIRA use, and observed that initial weight loss improved sperm concentration and count, with sustained effects for over one year among those maintaining weight loss.

In high-fat diet-fed mice, testosterone levels were reduced and not restored by GLP-1RA treatment; however, improvements were noted in sperm motility, mitochondrial function, and sperm DNA integrity (Zhang *et al*., 2015). Other animal studies also suggest that GLP-1 receptor activation may favorably affect spermatogenesis and testicular function (Jensterle *et al*., 2019). Additionally, a study of eugonadal men treated with dulaglutide for four weeks found no adverse effects on libido, hormone levels, or semen parameters (Lengsfeld *et al*., 2024).

Two recent clinical trials demonstrated positive effects of GLP-1RAs on reproductive function in obese men with functional hypogonadism (Gregorič *et al*., 2025; La Vignera *et al*., 2023). In the first, LIRA administered in escalating doses up to 3.0 mg/day over four months resulted in marked increases in total testosterone (+192.9%), SHBG (+157.1%), and gonadotropins (LH and FSH), indicating hypothalamic-pituitary-gonadal axis recovery. Significant reductions in body weight, BMI, HOMA-IR index, and abdominal circumference were also observed, along with substantial improvements in sperm motility (+142.9%) and erectile function. Compared with transdermal testosterone and gonadotropins, LIRA showed superiority by restoring eugonadism without suppressing the hormonal axis, representing a viable alternative for men desiring to preserve fertility (La Vignera *et al*., 2023).

Building on this evidence, Gregorič *et al*. (2025) conducted a 24-week RCT assessing semaglutide (1 mg/week) in men with type 2 diabetes, obesity, and functional hypogonadism. Treatment significantly increased normal sperm morphology (from 2% to 4%; *p*=0.012), sperm concentration and total sperm count, as well as total testosterone (+1.6 nmol/L) and clinical hypogonadism symptoms, with a mean weight loss of 6 kg. Unlike testosterone therapy, which suppresses LH and FSH and impairs sperm parameters, semaglutide preserved gonadal axis function (Gregorič *et al*., 2025).

Together, these studies support the hypothesis that GLP-1RAs provide metabolic and hormonal benefits and may also directly enhance spermatogenesis and sperm quality, given the presence of GLP-1 receptors on Sertoli and Leydig cells as well as spermatozoa. Thus, they emerge as promising therapeutic strategies for male infertility associated with obesity and functional hypogonadism.

## CONCLUSION

Obesity, especially when associated with PCOS, represents a significant factor in female infertility and an increasing public health challenge. In this context, GLP-1RAs, initially indicated for type 2 diabetes mellitus treatment, are gaining ground as effective agents for weight loss and more recently for their direct effects on female reproductive function (Duah & Seifer, 2025).

Clinical evidence demonstrates that GLP-1RAs, particularly LIRA and EXE, can significantly improve menstrual regularity, spontaneous ovulation, ovarian morphology, and pregnancy rates - even in patients with failure of first-line treatments. Combining these drugs with MET appears to potentiate such benefits, increasing rates of spontaneous or assisted pregnancy (Duah & Seifer, 2025).

Despite therapeutic advances in the preconception setting, important safety gaps remain regarding GLP-1RA use during pregnancy and lactation. Available human data are limited, generally observational or case reports, insufficient to guarantee fetal safety. Manufacturers themselves acknowledge the need for further long-term research. Thus, suspension of GLP-1RA therapy prior to conception and contraindication during breastfeeding are recommended (Gill & MacKey, 2021).

Regarding male fertility, findings remain inconclusive. While some studies suggest transient adverse effects on spermatogenesis, others report improvements in semen quality related to weight loss. Nonetheless, dedicated clinical trials in this population are needed.

Therefore, obstetricians and gynecologists play a strategic role not only in diagnosing and managing obesity in women but also in conscientiously prescribing and monitoring GLP-1RA use during reproductive years. Currently, many certified obesity medicine physicians are obstetrics and gynecology specialists - highlighting the importance of training these professionals who are often the sole physicians consulted by women of reproductive age (Gill & MacKey, 2021).

In summary, GLP-1RAs represent a promising pharmacological alternative for treating infertility associated with PCOS in overweight or obese women. When used judiciously, with appropriate contraceptive counseling and specialized follow-up, they can significantly improve reproductive outcomes. However, long-term reproductive safety, especially during pregnancy, requires robust and ongoing investigation.

## References

[r1] Abdalla MA, Deshmukh H, Atkin S, Sathyapalan T. (2021). The potential role of incretin-based therapies for polycystic
ovary syndrome: a narrative review of the current evidence. Ther Adv Endocrinol Metab.

[r2] Ammar OF, Sharma K, Liperis G, Fraire-Zamora JJ, Serdarogullari M, Ali ZE, Ramasamy R, Laurentino S, Watkins A, Mincheva M. (2023). Balancing the scales: the interplay of diet, exercise, GLP-1
receptor agonists, and obesity in shaping male reproductive
health. Hum Reprod.

[r3] Andersen E, Juhl CR, Kjøller ET, Lundgren JR, Janus C, Dehestani Y, Saupstad M, Ingerslev LR, Duun OM, Jensen SBK, Holst JJ, Stallknecht BM, Madsbad S, Torekov SS, Barrès R. (2022). Sperm count is increased by diet-induced weight loss and
maintained by exercise or GLP-1 analogue treatment: a randomized controlled
trial. Hum Reprod.

[r4] Bednarz K, Kowalczyk K, Cwynar M, Czapla D, Czarkowski W, Kmita D, Nowak A, Madej P. (2022). The role of GLP-1 receptor agonists in insulin resistance with
concomitant obesity treatment in polycystic ovary syndrome. Int J Mol Sci.

[r5] Burgart JM. (2020). Polycystic ovary disease and obesity: leptin, weight-loss
medication, and bariatric surgery. Clin Obstet Gynecol.

[r6] Burlina S, Dalfrà MG, Caprino R, Lapolla A. (2022). A case report on use of dulaglutide during the first weeks of
pregnancy in woman affected by type 2 diabetes mellitus. Acta Diabetol.

[r7] Cena H, Chiovato L, Nappi RE. (2020). Obesity, polycystic ovary syndrome, and infertility: a new avenue
for GLP-1 receptor agonists. J Clin Endocrinol Metab.

[r8] Cesta CE, Rotem R, Bateman BT, Chodick G, Cohen JM, Furu K, Gissler M, Huybrechts KF, Kjerpeseth LJ, Leinonen MK, Pazzagli L, Zoega H, Seely EW, Patorno E, Hernández-Díaz S. (2024). Safety of GLP-1 receptor agonists and other second-line
antidiabetics in early pregnancy. JAMA Intern Med.

[r9] Du Plessis SS, Omolaoye TS, Cardona Maya WD. (2024). Potential impact of GLP-1 receptor agonists on male fertility: a
fable of caution. Front Physiol.

[r10] Duah J, Seifer DB. (2025). Medical therapy to treat obesity and optimize fertility in women
of reproductive age: a narrative review. Reprod Biol Endocrinol.

[r11] Elkind-Hirsch K, Chappell N, Shaler D, Storment J, Bellanger D. (2021). A randomized, double-blind, placebo-controlled study of
liraglutide 3 mg (LIRA 3mg) on weight, body composition, hormonal and
metabolic parameters in women with obesity and polycystic ovary syndrome
(PCOS). Res Sq.

[r12] Elkind-Hirsch K, Marrioneaux O, Bhushan M, Vernor D, Bhushan R. (2008). Comparison of single and combined treatment with exenatide and
metformin on menstrual cyclicity in overweight women with polycystic ovary
syndrome. J Clin Endocrinol Metab.

[r13] Escobar-Morreale HF. (2018). Polycystic ovary syndrome: definition, aetiology, diagnosis and
treatment. Nat Rev Endocrinol.

[r14] Fontoura P, Cardoso MC, Erthal-Martins MC, Werneck C, Sartorio C, Ramos CF. (2014). The effects of liraglutide on male fertility: a case
report. Reprod Biomed Online.

[r15] Gill L, MacKey S. (2021). Obstetrician-gynecologists’ strategies for patient initiation and
maintenance of antiobesity treatment with glucagon-like peptide-1 receptor
agonists. J Womens Health (Larchmt).

[r16] Greco D. (2015). Normal pregnancy outcome after first-trimester exposure to
liraglutide in a woman with type 2 diabetes. Diabet Med.

[r17] Gregorič N, Šikonja J, Janež A, Jensterle M. (2025). Semaglutide improved sperm morphology in obese men with type 2
diabetes mellitus and functional hypogonadism. Diabetes Obes Metab.

[r18] Hall S, Isaacs D, Clements JN. (2018). Pharmacokinetics and clinical implications of semaglutide: a new
glucagon-like peptide (GLP)-1 receptor agonist. Clin Pharmacokinet.

[r19] Highlights MOUNJARO. (2025). Highlights of prescribing information.

[r20] Highlights SEMAGLUTIDE. (2025). Highlights of prescribing information.

[r21] Ivanišević M, Herman M, Horvatiček M, Lovrenčić MV, Đelmiš J. (2018). Pregnancy outcome and liraglutide levels in serum and umbilical
vein blood of a woman with type 2 diabetes: a case report. Gynaecol Perinatol.

[r22] Jeibmann A, Zahedi S, Simoni M, Nieschlag E, Byrne MM. (2005). Glucagon-like peptide-1 reduces the pulsatile component of
testosterone secretion in healthy males. Eur J Clin Invest.

[r23] Jensterle M, Janez A, Fliers E, Devries JH, Vrtacnik-Bokal E, Siegelaar SE. (2019). The role of glucagon-like peptide-1 in reproduction: from
physiology to therapeutic perspective. Hum Reprod Update.

[r24] Kalra S, Bhattacharya S, Kapoor N. (2021). Contemporary classification of glucagon-like peptide 1 receptor
agonists (GLP1RAs). Diabetes Ther.

[r25] La Vignera S, Condorelli RA, Calogero AE, Cannarella R, Aversa A. (2023). Sexual and reproductive outcomes in obese fertile men with
functional hypogonadism after treatment with liraglutide: preliminary
results. J Clin Med.

[r26] Lengsfeld S, Probst L, Emara Y, Werlen L, Vogt DR, Bathelt C, Baur F, Caviezel B, Vukajlovic T, Fischer M, Winzeler B. (2024). Effects of the glucagon-like peptide-1 receptor agonist
dulaglutide on sexuality in healthy men: a randomised, double-blind,
placebo-controlled crossover study. EBioMedicine.

[r27] Liu X, Zhang Y, Zheng SY, Lin R, Xie YJ, Chen H, Zheng YX, Liu E, Chen L, Yan JH, Xu W, Mai TT, Gong Y. (2017). Efficacy of exenatide on weight loss, metabolic parameters and
pregnancy in overweight/obese polycystic ovary syndrome. Clin Endocrinol (Oxf).

[r28] Maslin K, Alkutbe R, Gilbert J, Pinkney J, Shawe J. (2024). What is known about the use of weight loss medication in women
with overweight/obesity on fertility and reproductive health outcomes? A
scoping review. Clin Obes.

[r29] Merhi Z. (2025). Impact of dramatic weight loss with the new injectable
medications on reproduction in healthy non-polycystic ovary syndrome obese
women. F S Rep.

[r30] Minis E, Stanford FC, Mahalingaiah S. (2023). Glucagon-like peptide-1 receptor agonists and safety in the
preconception period. Curr Opin Endocrinol Diabetes Obes.

[r31] Muller DRP, Stenvers DJ, Malekzadeh A, Holleman F, Painter RC, Siegelaar SE. (2023). Effects of GLP-1 agonists and SGLT2 inhibitors during pregnancy
and lactation on offspring outcomes: a systematic review of the
evidence. Front Endocrinol (Lausanne).

[r32] Nuako A, Tu L, Campoverde Reyes KJ, Chhabria SM, Stanford FC. (2023). Pharmacologic treatment of obesity in reproductive aged
women. Curr Obstet Gynecol Rep.

[r33] Nylander M, Frøssing S, Clausen HV, Kistorp C, Faber J, Skouby SO. (2017). Effects of liraglutide on ovarian dysfunction in polycystic ovary
syndrome: a randomized clinical trial. Reprod Biomed Online.

[r34] Papaetis GS, Kyriacou A. (2022). GLP-1 receptor agonists, polycystic ovary syndrome and
reproductive dysfunction: current research and future
horizons. Adv Clin Exp Med.

[r35] Pasquali R, Gambineri A. (2018). New perspectives on the definition and management of polycystic
ovary syndrome. J Endocrinol Invest.

[r36] Sacha CR, Page CM, Goldman RH, Ginsburg ES, Zera CA. (2018). Are women with obesity and infertility willing to attempt weight
loss prior to fertility treatment?. Obes Res Clin Pract.

[r37] Salamun V, Jensterle M, Janez A, Vrtacnik Bokal E. (2018). Liraglutide increases IVF pregnancy rates in obese PCOS women
with poor response to first-line reproductive treatments: a pilot randomized
study. Eur J Endocrinol.

[r38] Saxenda review (2025). Saxenda patient labeling review. FDA;.

[r39] Service CA, Puri D, Al Azzawi S, Hsieh TC, Patel DP. (2023). The impact of obesity and metabolic health on male fertility: a
systematic review. Fertil Steril.

[r40] Sola-Leyva A, Pathare ADS, Apostolov A, Aleksejeva E, Kask K, Tammiste T, Ruiz-Durán S, Risal S, Acharya G, Salumets A. (2025). The hidden impact of GLP-1 receptor agonists on endometrial
receptivity and implantation. Acta Obstet Gynecol Scand.

[r41] Sundararaman L, Gouda D, Kumar A, Sundararaman S, Goudra B. (2025). Glucagon-like peptide-1 receptor agonists: exciting avenues
beyond weight loss. J Clin Med.

[r42] Wang J, Johnson T, Sahin L, Tassinari MS, Anderson PO, Baker TE, Bucci-Rechtweg C, Burckart GJ, Chambers CD, Hale TW, Johnson-Lyles D, Nelson RM, Nguyen C, Pica-Branco D, Ren Z, Sachs H, Sauberan J, Zajicek A, Ito S, Yao LP. (2017). Evaluation of the safety of drugs and biological products used
during lactation: workshop summary. Clin Pharmacol Ther.

[r43] Williams J, Pomeroy NE, Pop-Busui R, Lash R, Douyon L, Chames M, Wyckoff J. (2009). Case report: Exenatide use during pregnancy. Endocrinologist.

[r44] Yang Q, Wang F. (2016). Successful pregnancy after improving insulin resistance with the
glucagon-like peptide-1 analogue in a woman with polycystic ovary syndrome:
a case report and review of the literature. Gynecol Obstet Invest.

[r45] Zhang E, Xu F, Liang H, Yan J, Xu H, Li Z, Wen X, Weng J. (2015). GLP-1 receptor agonist exenatide attenuates the detrimental
effects of obesity on inflammatory profile in testis and sperm quality in
mice. Am J Reprod Immunol.

[r46] Zhao M, Liao B, Yun C, Qi X, Pang Y. (2024). Liraglutide improves follicle development in polycystic ovary
syndrome by inhibiting CXCL10 secretion. Reprod Biol Endocrinol.

[r47] Zhou L, Qu H, Yang L, Shou L. (2023). Effects of GLP1RAs on pregnancy rate and menstrual cyclicity in
women with polycystic ovary syndrome: a meta-analysis and systematic
review. BMC Endocr Disord.

